# Innovation drivers of external competitiveness in the great recession

**DOI:** 10.1007/s11187-021-00453-0

**Published:** 2021-01-29

**Authors:** Emanuele Brancati, Raffaele Brancati, Dario Guarascio, Antonello Zanfei

**Affiliations:** 1 Deparment of Economics and Law, Sapienza University of Rome, Rome, Italy; 2 MET, Rome, Italy; 3 University of Urbino ‘Carlo Bo’, Urbino, Italy

**Keywords:** SMEs, Competitiveness, Internationalization, Innovation, R&D, D22, F00, O3, L26

## Abstract

This paper explores the main drivers of firms’ external competitiveness in times of crisis. We focus on the aftermath of the Great Recession (2008–2015) and present evidence based on a comprehensive survey of Italian companies (the MET dataset). Overall, our results highlight not only the strict correlation between internationalization and innovative activities but also a positive change of attitude of Italian firms towards these strategies. We show that, while structural factors play a key role for external competitiveness, other critical aspects trigger superior performances, especially strategic profiles, technological capabilities, and proactive behaviors such as innovativeness and R&D investment. Importantly, we document disproportionate effects of innovation for smaller and less productive companies. This points at dynamic strategies as a potential tool to fill the gap between larger/more productive companies and the set of less structured firms, a segment representing an ideal target for policy measures.

**Supplementary Information:**

The online version contains supplementary material available at 10.1007/s11187-021-00453-0.

## Introduction

During rainy days, both policy makers and analysts focus their attention on those paying the highest price: companies kicked out of the market and workers losing their jobs accordingly. Such a focus is perfectly justified by the need of setting up policies aimed at minimizing the number of businesses going bankrupt and healing the wounds from depression, so to downsize the overall economic consequences. However, identifying firms that are resilient to the unfolding of a crisis eventually growing in both national and international markets is an equally important task. Companies successfully resisting and reacting to a negative shock are in fact those capable of adjusting their strategy (and organizational set-up) to a fast-evolving context so to cushion the effect in the short-run and enjoy a competitive advantage as soon as the economy recovers. At the macro-level, the speed of recovery is in fact crucially linked to the amount of healthy firms that can help in pulling the rest of the economy out of the swamp.

When a large crisis outbreaks, the collapse of aggregate demand is one of the major threats to firms’ survival, especially for companies prevalently serving domestic markets. In the aftermath of financial and sovereign debt crisis, the domestic component of aggregate demand tended to fall relatively more vis-à-vis the international one. This is particularly true in contexts wherein the public component of domestic demand is depressed by a deflationary (i.e., austerity) policy set-up, as the one followed by the Southern European economies after the 2008 crisis.^1^ In such economies, both public spending and private consumption shrank as a consequence of the crisis and of the ensuing need to reduce the debt exposure prompted by the financial crisis. Within this context, consolidating positions in international markets, or penetrating into new ones, represented not only a key way to survive in the middle of a crisis but, in some cases, a strategy to profit out of it, eventually growing at the expenses of competitors.

When crises have a global scale, firms that keep on exporting are likely to gain new market shares left by other companies in distress. Besides, the existence, emergence, and growth of resilient firms and the identification of their characteristics can eventually pave the way to the emulation of other companies which might reinforce the overall improvement in productivity and economic performance in general. Policy makers should thus pay a particular attention to the key drivers (internal and external to the firm) which might explain a positive performance during bad times, and create the conditions that are conducive to external competitiveness. From this perspective, their task appears to be twofold: favoring healthy companies in undertaking their activities in foreign markets and helping fragile firms to develop the key capabilities underlying a greater competitiveness.

When SMEs represent the dominant share of the industrial structure, the attention on those displaying resilience and dynamism is even more important. Often financially constrained and lacking the necessary technological and organizational capabilities, SMEs are more likely—as compared to large companies—to fail in trying to meet markets where demand continues to be dynamic despite the crisis (Coad 2009; Brancati et al. 2018). On the other hand, profiling (i.e., identifying key characteristics and performance’s determinants) and supporting those SMEs capable to adjust their strategies and grow even in times of crisis represent crucial policy tasks in economies where firms are prevalently small. In fact, due to their tight linkages with the local economy (including other SMEs operating in the same area/sector), resilient SMEs represent a critical asset to avoid the unfolding of a wider and deeper recession. In this regard, being capable to internationalize and/or to intensify the presence in foreign markets represent factors that, above others, can explain SMEs’ resilience and growth. This is especially true when domestic demand shrinks as a consequence of a crisis (Coad 2009; Motta 2020).

This work contributes to the empirical literature on the determinants of external competitiveness for SMEs. Our focus on the Italian economy in the 2008–2015 period allows us to identify the main drivers of resilience in times of great distress. Indeed, the aftermath of the 2008 financial crisis and the unfolding of the sovereign-debt crisis brought about structural effects on industries, markets, as well as on companies’ attitudes and behavior. In Italy, the crisis was particularly severe causing a drop in aggregate production of about 25% (Lucchese et al. 2016; Cirillo et al. 2017; Dosi et al. 2019). In this context, the ability to keep (or to gain) international market shares proved to be the main way to survive and, in some cases, to improve the performance of Italian firms. Because its industrial structure is predominantly composed of SMEs—most of which micro-sized—Italy represents an instructive laboratory to investigate the drivers of SMEs’ international competitiveness and growth.

Our study relies on one of the largest firm-level surveys administrated in a single European country, the MET (Monitoraggio, Economia e Territorio) dataset, providing a wide set of information on all Italian companies, including micro-sized companies with less than ten employees. Our sample is largely composed of SMEs with an average size of 8 employees (96% of firms below 250 employees, 85% below 20). This is an essential feature of our analysis as micro-firms represent the vast majority of the Italian population and the segment of the market that is, a priori, more fragile in times of recession.

The empirical strategy employs random-effect probit models (with Mundlak correction) and within estimator (with firm and time fixed effects) to correct for unobserved heterogeneity and dig deeper into what drives gains in external competitiveness. As expected, we confirm a self-selection mechanism of more productive companies into international markets. However, once accounted for persistent components of heterogeneity, underlying long-lasting differences in firm efficiency, the residual role of productivity is found to be significantly reduced in explaining the change in the exporting status. Gains in external competitiveness are found to be strictly linked to firms’ strategic behavior and investment in dynamic strategies. In particular, firms’ innovativeness and involvement in R&D projects are found to induce a significant positive effect on export strategies and performance.

In this regard, we explore several dimensions of heterogeneity by showing the existence of compounding (cumulative) impact of dynamic strategies and emphasizing the role of upgrading/downgrading paths (i.e., whether the behavior in the recent past consisted in an increase or decrease in the array of strategies adopted, see Section 5.2 for a detailed discussion).

Among the different forms of innovations, new products dominate other forms of innovativeness (process or organizational-managerial), especially in case of previously non-exporting companies. However, we find process and organizational innovations to have an additional effect on export by boosting firms’ long-run productivity, which represents an additive indirect effect of innovation on external competitiveness.

Importantly, although our results are all based on a sample mainly composed of SMEs, we further shed light on significant heterogeneities across firms according to their size and characteristics. In particular, innovative strategies are found to have disproportionate effects (3-to-4 times larger) for the international performance of (originally) smaller and less productive companies. This is an important result as it points at innovativeness as a potential tool to fill the gap between larger and more productive companies and the set of less structured firms, a segment that represents an ideal target for policy measures.

The remainder of the paper is the following. Section 2 provides a review of the literature dealing with the drivers of companies’ external competitiveness (with a specific focus on SMEs) and spells the research questions upon which the empirical analysis is built. Section 3 illustrates the data and some descriptive evidence concerning the key variables at stake. Section 4 presents the econometric strategy while Section 5 discusses the results. Finally, Section 6 concludes the paper and provides some policy remarks.

## Background literature

This section reviews the empirical literature on the drivers of external competitiveness. First, we briefly go through the strand of research investigating firm-level determinants of external competitiveness. We then provide a synthetic review of the studies specifically studying SMEs and, finally, we offer an ad-hoc focus on the Italian case.

### Firm level determinants of external competitiveness

A vast empirical literature attempted to identify the determinants of companies’ external competitiveness. However, univocally identifying drivers (and companies’ characteristics) laying behind successful performances on international markets is all but an easy task. Three largely interdependent elements are at the forefront: cost, technology, and firm-level heterogeneities (Laursen and Meliciani 2010; Dosi et al. 2015; Bogliacino et al. 2017). A classical path followed by firms to penetrate international markets (or to increase their market shares) is to reduce costs and increase the attractiveness of their goods and services in terms of relative prices. This can clearly be done by either resorting to cheaper inputs or making production more efficient via process innovation (Pianta and Vivarelli 2000). Nevertheless, consistent with a Schumpeterian view of innovation-based competition, a large bulk of the literature reported that technological and product quality–related factors are substantially more relevant in explaining export performances than cost-related ones. Since the seminal contribution of Vernon (1966), product innovation has been identified as a fundamental key to enter foreign markets. Young innovative firms rely on exports to (i) increase their market shares, (ii) enjoy demand-pull effects (Deschryvere 2014), and (iii) accumulate the capital needed to further develop their products (Antràs 2005). Reversing the perspective, technological competitiveness strategies based on innovations are more likely to characterize exporting firms rather than those prevalently serving domestic markets. In fact, a superior technology is likely to be needed to access foreign markets characterized by a fiercer competition based on quality and technology (together with a wider and variegated spectrum of consumer preferences). Cassiman et al. (2010) suggest that the introduction of new products might induce a positive productivity shock allowing firms to access and improve their position in international markets. As emphasized by Foster et al. (2008), successful new products are likely to stimulate firm-specific positive demand shocks activating a “virtuous circle” going from innovation to productivity and resulting in greater export-market shares (Cantwell and Sanna Randaccio 1990). This mechanism matches with the “learning-by-exporting” hypothesis (De Loecker 2007, 2013). The latter, again, is linked to the larger and more variegated number of customers and competitors that companies might face but, also, to the greater opportunities in terms of imitation and networking they are likely to seize. In all these cases, firm-specific capabilities are expected to increase with positive effects on productivity and export performance. Empirical evidence confirming this hypothesis abounds.^2^

If new products are widely recognized as a key driver of export success (Coad 2009), the recipe should not be thought to work indiscriminately for all firms (Guarascio and Tamagni 2019). An extensive literature has in fact reported the presence of substantial and persistent heterogeneity along virtually every dimension of firm performance (Bartelsman and Doms 2000; Castellani and Zanfei 2007). This heterogeneity also characterizes the restricted cluster of exporting firms, as confirmed by Bernard and Jensen (2004), Greenaway and Kneller (2007), and Mayer et al. (2014).

Contrasting with the hypothesis of a “healthy cleansing” role of recessions (Foster et al. 2016), the 2008 crisis has further increased such firm-level heterogeneity. On the one hand, a cluster of dynamic (mostly exporting) companies displayed resilience and manage to grow out of the crisis. On the other, an even larger group of low-productive firms managed to survive, despite a persistently sluggish performance, pull down the overall economy productivity (Dosi et al. 2019). The survival of these “zombie firms” (Adalet McGowan et al. 2017a and 2017b), combined with the exit of footloose foreign firms (Varum et al. 2014), might partly explain the low-productivity dynamic observed in several countries in the post-2008 recession.

### Explaining SMEs’ export performance

In many countries, SMEs represent the dominant segment of the market. This is particularly true in Europe, with Italy and Spain registering values close to or greater than 90% of total enterprises. In such economies, SMEs provide a substantial contribution to aggregate growth, in terms of both value added and employment (Ayyagari et al. 2007, 2014). Nevertheless, information asymmetries, financial constraints, and scarcity of (physical and human) capital might hinder SMEs from expanding their business operations (Berger and Udell 2006). Being relatively less capital intensive, SMEs may face difficulties in accessing bank financing (Beck and Demirguc-Kunt 2006). Such a combination of low capitalization and reduced access to external funds might result in a lower investment propensity of SMEs as compared to larger companies (Chavis et al. 2011).

If, on the one hand, SMEs might be relatively more vulnerable in the midst of a crisis, on the other hand, their size and flexibility may allow them to be comparatively faster and effective in adapting to changing economic contexts. In other words, SMEs are characterized by an even higher degree of heterogeneity than larger companies. According to the traditional “Schumpeterian” distinction between large Mark II– and small Mark I–type of firms (Breschi et al. 2000), the latter can be dynamic enough to “creatively destroy” existing market structures through the introduction of innovative products and new organizational modes. Large firms, in turn, may lack the flexibility necessary to adapt to changes in the market context. In this regard, Varum and Rocha (2013) find that larger companies suffer higher negative effects of economic downturns on firm growth, both during and immediately following periods of crisis, and suggest that SMEs may act as potential stabilizers in downturn periods. Concerning the innovation strategies that SMEs tend to adopt, Acs and Audretsch (1990), Audretsch and Vivarelli (1996), and Conte and Vivarelli (2014) emphasize how, given the financial and technological constraints they face, SMEs are more likely “to buy” rather than “to make” innovations (i.e., technological acquisitions). This is mostly due to the lack of (technological and financial) internal resources that are required to develop large R&D projects.^3^ On the other hand, a recent empirical literature (see, for a review, Pellegrino and Piva 2020) has emphasized the key role that young and small innovative firms can play in driving innovation in sectors characterized by lower appropriability conditions and higher technological opportunities (i.e., “entrepreneurial sectors”). However, despite SMEs are less likely to conduct formal R&D than larger firms, their relative efficiency (i.e., number of patents and innovations by unit of R&D input) in performing R&D can be higher (see for instance Acs and Audretsch 1990; Conte and Vivarelli 2014).^4^

In times of crisis, especially if the growth of domestic demand is lower than in international markets, SMEs displaying a low propensity towards change and diversification are more likely to face insolvency and bankruptcy. Conversely, export-oriented SMEs relying on dynamic strategies are more likely to be endowed with the technological, organizational, and managerial capabilities that might guarantee resilience and growth (Salavou et al. 2004; Coad 2009). In this regard, idiosyncratic characteristics such as “proactivity” and attitudes towards changes in business strategies are of paramount importance in explaining SMEs’ external competitiveness. This is, once again, especially true in times of crisis, when opportunities shrink and competition becomes harsher.

### The Italian case

In Italy, SMEs represent the backbone of the industrial structure contributing, on both national and international markets, to the overall economy’s performance. Having access to unique data covering the whole Italian industrial structure including micro-firms (a large but often empirically neglected component of the universe of Italian SMEs), we are thus in the condition of investigating SMEs’ competitiveness determinants at a high level of detail as compared to the previous literature. In Italy, the heterogeneity characterizing this segment of the market is, however, substiantial. Coexisting with a dynamic, innovative, and export-oriented bulk of SMEs, there is an even larger cluster (in terms of size and employment weight) characterized by low productivity and weak growth performance (Bogliacino et al. 2017). After the 2008 crisis, the width of inter-firm productivity distributions has expanded even further (see, for instance, Dosi et al. 2019).

One of the key elements laying at the roots of such persistent heterogeneity is the distinction between domestic and export-oriented firms (on this point, see, among the others, Castellani and Zanfei 2007; D'Aurizio and Cristadoro 2015). Focusing on the determinants of export success, Basile (2001) finds a pivotal role played by innovation (new products, in particular) while cost factors—as labor costs per unit of product—seem to play a more marginal role. In a similar vein, Barba Navaretti and Castellani (2004) compare productivity dynamics across different firm categories, and find that internationalized companies substantially outperform domestic businesses. These results are further confirmed by Serti et al. (2010), and Castellani and Giovannetti (2010), among others.

Adopting an evolutionary approach, other studies (Dosi and Nelson 2010) link the persistent heterogeneity characterizing Italian companies’ international performance to their idiosyncratic features concerning knowledge base, organizational routines, and business strategies. On the other hand, a large set of contributions put emphasis on Foreign Direct Investments (FDIs), multinational companies (MNCs), and offshoring strategies (Barba Navaretti and Castellani 2004; Cozza and Zanfei 2014).^5^

We contribute to this broad literature by presenting novel evidence on the drivers of firms’ export performance in times of crisis, with a special focus on SMEs. We explore several dimensions of external competitiveness, ranging from extensive margins of export to intensive margins, as well as entry/exit in/from international markets. We emphasize the importance of behavioral and strategic factors by looking at both their direct and indirect effects on export, and specifically model several dimensions of heterogeneity showing disproportionate effects for (ex ante) smaller and less productive companies. More in detail, we add to three distinct strands of literature: (i) the one focusing on the determinants of firm-level performance (for a review, see Audretsch et al. 2014), (ii) the one dealing with the heterogeneous distribution of performance indicators and exploring the company-level idiosyncratic characteristics which may lay behind such heterogeneity (Dosi et al. 2012), and (iii) the empirical one investigating the peculiar characteristics of the Italian economy with an emphasis on the role of SMEs (Brancati et al. 2018).

Building upon the above literature, the first research question we aim to address can be spelled out as follows:

- RQ1—*Which are the key drives of companies’ success in international markets?*

The aim of our first research question is to open the “black box” of companies’ external competitiveness, the latter being measured in terms of exports’ extensive margin. First, we focus on the role exerted by the more standard and comprehensive performance indicator: labor productivity. Besides, we explicitly account for the whole set of innovation strategies (i.e., product, process, and organizational innovation) that are also capable, above and beyond productivity, to affect international performance. The analysis is performed controlling for the large amount of firm-level factors (i.e., size, age, capitalization, previous record in terms of internationalization) that are also likely to affect companies’ competitiveness.

To go some steps further, we put at center stage the direct and indirect relationships between innovation, productivity, and international performance (Griliches 1979; Crépon et al. 1998; Bogliacino et al. 2017). That is, we test whether different types of innovations can have an *indirect effect* on companies’ external performance by affecting the growth of productivity. Therefore, our second research question turns out to be the following:

- RQ2—*How do different types of innovation (i.e., product, process, and organizational) impact on companies’ productivity?*

Concerning the measurement of external competitiveness, we extend the focus on the intensive margin of exports. By focusing on both export shares and growth of exports, we verify whether being a relatively more productive firm ensures, all things being equal, a consolidation of their international positioning also to companies that are already exporting. As a result, RQ3 states:

- RQ3—*Does productivity have an impact on the intensive margin of companies’ international performance?*

Finally, the role of productivity and of its innovative determinants is further investigated to test if and to what extent any disproportionate effect on micro- and less-productive firms can be detected. In other words, we aim at verifying whether adopting productivity-enhancing strategies represents a way to “catch-up” in terms of external competitiveness for companies lacking relevant technological and financial resources (i.e., micro-firms) or having poor productivity performance (i.e., companies that at the beginning of the sample are below the 25th percentile of the respective cross-sectional distribution). The fourth RQ is thus formalized as follows:

- RQ4—*Do innovative activities (i.e., R&D efforts and innovative output including both product and process innovation) have a disproportionate effect on micro- and relatively less-productive companies’ external competitiveness?*

In the following section, we illustrate the data adopted to test the above-mentioned RQs and provide some descriptive evidence concerning the key variables at stake. Subsequently, we present the econometric strategy and discuss the related results.

## Data

Our main source of information is 2008–2015 MET (Monitoraggio, Economia e Territorio) database on the Italian industry, one of the widest surveys administrated in a single European country. The dataset is made of roughly 25,000 observations per wave (six in total) and provides information on a rich set of firms’ strategic profiles such as innovation, R&D, and networking relationships. This survey is specifically conceived to study a massive amount of firms’ characteristics and strategies, with a particular focus on their internationalization and innovative patterns. The sample is representative of Italy’s population of firms along three dimensions: 2-digit sectors (manufacturing and production services only), region, and size class.^6^

Importantly, unlike most other firm-level databases, the MET survey also accounts for micro-sized firms with less than ten employees representing the vast majority of companies within the Italian industrial system. This characteristic is essential to our purpose of analyzing the role of innovative strategies in affecting external competitiveness and overcoming ex ante fragilities such as size and productivity disadvantages. The availability of a large amount of information on SMEs and on micro-firms allows us to explore in depth the determinants of their competitive performances as well as the heterogeneity detectable among them.

Survey data are then matched with official balance-sheet information from CRIF-Cribis D&B, with a final estimation sample that ranges between 50,000 and 13,000 observations.

Descriptive statistics are presented in Table 1. Roughly 29% of the companies declare to sell their products to international markets, accounting for an average of 9% of total revenues from exported products. As for firms’ innovativeness, 31% of the firms introduced at least one type of innovation in the previous year (18% product innovations, 15% process innovations, and 17% organizational-managerial innovations) while 16% of the companies invested in R&D programs, with an average expenditure of 5% of total sales. Conditional averages show a strong positive correlation between firm innovative activities and export status. Innovators have twice the probability of exporting with respect to non-innovators (22% vs 44%) and three-times the share of sales from exported products (6% vs 17% of total sales). Similarly, firms involved in R&D projects are significantly more exposed to international markets both in terms of extensive (23% vs 63%) and intensive margin of export (5% vs 26%).

**Table 1:** Descriptive statistics

	Mean	Stdev	Min	Max
Export	0.287	0.453	0	1
Export share	9.893	21.46	0	100
Export sales growth	0.005	0.491	−1	2.553
Export sales	18.51	2.161	1.609	26.88
Productivity	10.63	1.308	−16.13	26.38
Innovation	0.314	0.464	0	1
Product inn.	0.188	0.391	0	1
Process inn.	0.148	0.355	0	1
Organiz. inn.	0.168	0.374	0	1
R&D	0.162	0.369	0	1
R&D share	1.035	5.217	0	250
Size	2.272	1.742	−11.51	10.72
Age	2.544	2.113	−13.81	6.405
Capitalization	9.451	3.941	−21.06	27.51
Group	0.129	0.335	0	1
Group multinational	0.026	0.160	0	1
Import	0.113	0.316	0	1
Investment	0.445	0.497	0	1
ROA	-0.001	0.104	−0.619	0.266
Vertical int.	0.363	0.225	0.026	1.197
Cost of labor	0.251	0.229	−22.42	62.44
Sales	1.162	6.722	0	3675
Sales growth	−0.031	0.563	−14.17	10.78
Leverage	1.649	1.089	−1.822	12.19
Net acc. payable	−0.101	0.338	−2.385	86.71
Bank debt	0.252	0.402	−2.307	73.33

## Economic strategy

The econometric strategy aims at analyzing several dimensions of firms’ external competitiveness, ranging from probabilities of export, entrance, or exit, to international performance and productivity dynamics. The baseline equation tests the effect of different drivers of external competitiveness on the extensive margins of export (i.e., probability of exporting) according to the following specification:

1 $$ \Pr \left({Y}_{it}=1\right)=\Phi \left({\beta}^{\prime }{Z}_{it-1}+{\lambda}_t+{c}_i+{\varepsilon}_{it}\right) $$

where *Y*_*it*_ is either a dummy-dependent variable identifying export activity (*Export*), or other dimensions of firms’ internationalization. A continuous dependent variable will be used when intensive margins are considered. Equation 1 is a standard reduced form including a rich set of regressors (*Z*_*it* − 1_) to capture structural characteristics (productivity, size, age, capitalization, group belonging and whether the group is a multinational one, degree of vertical integration, cost of labor), financial characteristics (leverage, trade credit, bank debt, ROA, sales, cash flow), and especially firms’ strategic behavior (innovativeness, R&D, investments, networking, import propensity, and human capital intensity). All regressors are lagged once so to avoid simultaneity bias (see below). We also include time effects (*λ*_*t*_) to capture common shocks and cyclical components that vary over time. Finally, *c*_*i*_ is a factor controlling for firms’ unobserved heterogeneity, also accounting for permanent industrial/geographical effects (12 controls for firms’ industry belonging and 107 for geographical provinces).^7^

There are two main issues we need to take into account in order to assess the effect of different drivers on firms’ external competitiveness. The first one has to do with reverse causality, whereby characteristics and behaviors do not foster export performance but instead result from the (a priori) successful penetration into new markets. The second interrelated point is the self-selection of more productive and dynamic companies into international environments. Because of the lack of a natural experiment allowing us to define a strictly exogenous set of instruments, we address these issues in several alternative ways.

First of all, we rule out simultaneity bias by matching current export status with lagged regressors to alleviate problems of reverse causality. Furthermore, we account for unobserved heterogeneity in a binary response framework by employing random-effect probit models augmented with the time average of each regressor (i.e., Mundlak-type controls). As suggested by Wooldridge (2010), we relax the unrealistic assumptions of random-effect models (the orthogonality of *c*_*i*_ with respect to the full set of regressors) by focusing on the effect of each variable in terms of deviations from its time average. This allows us to purge the model from persistent heterogeneity across firms that may lead to biased estimates. Moreover, to further control for persistence of *Y*_*it*_, we also provide results for the subset of firms with *Y*_*it* − 1_ = 0.

In the unlikely case that residual heterogeneity is still affecting our findings, we run several additional robustness checks. We account for the introduction of unrestricted firm fixed effects through linear probability (within estimators) models taking into account all the firms’ characteristics that are stable over time. Finally, we further take care of self-selection by employing matching techniques (Coarsened Exact Matching) and control for correlated shocks in the Great Recession by enriching our baseline specifications with an extensive set of time-fixed effects specific for firm belonging industry (12 macro-industries × 5 periods) and geographical province (110 × 5).

## Results

This section presents the results of the analysis. The estimates are divided in two blocks. The first one reports the result for the whole sample of Italian companies included in the analysis. The second one explicitly focuses on micro-sized companies and “fragile” firms. In both cases, we start exploring direct and indirect effects of dynamic strategies on the firms’ probability of export (i.e., extensive margins). We then analyze heterogeneities in the effect and present results on the intensive margins to emphasize the role of innovative strategies as an instrument for overcoming size and productivity disadvantages.

### Entire economy

#### Extensive margins: baseline

Columns 1 and 2 of Table 2 present some preliminary evidence based on the pooled probit estimators (marginal effects with robust standard errors are reported). Even though this approach neglects firms’ unobserved heterogeneity and reverse causality issues, it may still provide useful guidance in the establishment of clean correlations between exporting status and firms’ characteristics and behaviors.

**Table 2:** Extensive margins of export

Y:	Export					
Estimator:	Pooled probit		RE-Probit with Mundlak		Linear probability model	
	(1)	(2)	(3)	(4)	(5)	(6)
Productivity	0.0281***	0.0208***	-0.001	0.0011	0.006*	0.008
	(0.0022)	(0.0046)	(0.002)	(0.002)	(0.003)	(0.011)
Size	0.058***	0.056***	0.017***	0.031***	0.021***	0.053***
	(0.001)	(0.003)	(0.003)	(0.004)	(0.004)	(0.012)
Age	−0.003	−0.009**	−0.0012	−0.002	−0.000	−0.002
	(0.002)	(0.004)	(0.002)	(0.002)	(0.003)	(0.006)
Capitalization	0.001	-0.006***	0.001	0.002	0.000	0.005
	(0.001)	(0.002)	(0.001)	(0.002)	(0.001)	(0.004)
Group	0.008	0.006	0.009	0.0100	0.014	0.004
	(0.009)	(0.008)	(0.006)	(0.009)	(0.010)	(0.018)
Group multinational	0.179***	0.167***	0.073***	0.070***	0.075***	0.095**
	(0.024)	(0.026)	(0.025)	(0.026)	(0.031)	(0.044)
Import	0.341***	0.332***	0.080***	0.071***	0.200***	0.175***
	(0.006)	(0.011)	(0.004)	(0.005)	(0.006)	(0.012)
Innovation	0.053***	0.052***	0.011**	0.013***	0.016***	0.018*
	(0.004)	(0.007)	(0.0045)	(0.005)	(0.005)	(0.009)
R&D share	0.004***	0.004***	0.0021***	0.0021***	0.003***	0.003***
	(0.000)	(0.000)	(0.001)	(0.001)	(0.000)	(0.001)
Investment	0.013***	0.010	0.014***	0.015***	0.025***	0.023**
	(0.004)	(0.007)	(0.0045)	(0.005)	(0.005)	(0.010)
ROA	−0.019***	−0.011*	−0.007	−0.007	−0.001	0.034
	(0.003)	(0.005)	(0.045)	(0.045)	(0.011)	(0.088)
Vertical int.	0.0108**	0.017**	0.0122	0.0116	−0.019	−0.017
	(0.004)	(0.009)	(0.017)	(0.029)	(0.017)	(0.054)
Cost of labor	0.028	-0.041	0.0112	0.0257	0.001	0.090
	(0.019)	(0.056)	(0.029)	(0.045)	(0.004)	(0.089)
Sales	−0.253***	−0.222***	--	−0.005	--	−0.001
	(0.011)	(0.0325)	--	(0.007)	--	(0.012)
Sales growth	−0.004	−0.074*	--	−0.0027	--	−0.009
	(0.004)	(0.0404)	--	(0.005)	--	(0.009)
Leverage	--	−0.0272***	--	−0.0020	--	0.003
	--	(0.004)	--	(0.0044)	--	(0.008)
Net acc. payable	--	0.112***	--	0.0105	--	0.008
	--	(0.0211)	--	(0.022)	--	(0.040)
Bank debt	--	0.102***	--	−0.043*	--	−0.126**
	--	(0.024)	--	(0.024)	--	(0.048)
Constant	−1.588***	−0.825***	−2.55***	−2.88***	0.18***	0.09
	(0.092)	(0.193)	(0.244)	(0.368)	(0.047)	(0.150)
Controls						
Region		Yes	Yes	Yes	Yes	Yes
Industry		Yes	Yes	Yes	Yes	Yes
Time		Yes	Yes	Yes	Yes	Yes
Region*Time		No	No	No	No	No
Industry*Time		No	No	No	No	No
Mundlak		Yes	Yes	Yes	--	--
Firm FE		--	--	--	Yes	Yes
Observations	41.756	14.318	23.932	14.318	55.441	15.327
Pseudo *R*^2^	--	--	0.757	0.765	--	--
*R*^2^	0.249	0.272	--	--	0.059	0.042

Pooled probit (marginal effects in columns 1–2), RE-probit models with Mundlak correction (marginal effects in columns 3–4), and linear probability models with firm and time fixed effects (columns 5–6). The dependent variable is the extensive margin of export (Export). All measures are defined in the Appendix

*, **, *** denote, respectively, significance at 10%, 5%, and 1% level

Robust standard errors in parentheses

In response to RQ1, there are several issues that are worth mentioning. First, we find that more productive companies are largely associated to higher probabilities of export: a one-standard deviation increase in productivity is associated to a 2–2.8% higher probability of export. This evidence is broadly in line with the dominant literature on firms’ internationalization. However, given the type of econometric analysis performed that neglects persistent heterogeneity across firms, little can be said on whether this effect is linked to reverse causality, the ex ante self-selection of more productive companies into international markets, or learning by exporting phenomena. The role of productivity on firms’ probability of export will be further discussed in the following analyses.

Second, structural characteristics play a critical role for firms’ exporting status. In particular, firm size and the belonging to corporate groups—only if multinational—are associated to increases in a company’s probability of exporting.^8^ On the other hand, older and more capitalized firms are found to have a lower international propensity, even though coefficients tend to be unstable across specifications.

A prominent effect is found for firms’ strategic behaviors. Being an importer of intermediate products is largely associated to a higher probability of exporting (33–34% probability). This result is in line with the extant literature emphasizing how import policies may affect aggregate productivity, resource allocation, and industry export activity along both the extensive and intensive margins (see for instance Amiti and Konings 2007; Halpern et al. 2015; Kasahara and Lapham 2013; Kasahara and Rodrigue 2008).

Importantly, the introduction of innovations and the involvement in R&D projects, as well as undertaking new investments, are strongly correlated with export strategies, thus providing some initial elements to tackle RQ2 on the role of innovation strategies in export performance. At this stage though, the estimates do not allow to make inference on the direction of the nexus underlying these relationships.

As expected, past performances are positively related to firms’ exporting status, but, once again, this analysis does not shed light on whether the direction of causality is reversed (i.e., the access to international markets allowed firms to experience higher sales growth). Other estimates are in line with the extensive literature reviewed in Section 2 on innovation as key drivers of export competitiveness.

Since these results may be affected by persistent unobserved heterogeneity across firms (such as managers’ attitudes and skills), Table 2 also presents RE-probit models with Mundlak correction (columns 3 and 4) and within estimators with firm and time-fixed effects (columns 5 and 6).

Once purged the model from persistent heterogeneity across firms, the role of productivity in fostering export activity is found to be strongly reduced. This result is unchanged across the two estimators and is qualitatively similar if we employ TFP as an alternative measure of productivity (Levinsohn and Petrin 2003). Notice that the comparison of columns 2 and 4 (or 6) is implicitly confirming the self-selection hypothesis (largely emphasized by the literature), whereby persistently more productive companies are more prone to penetrate international markets, driving the positive association in the pooled specification. However, once this persistent heterogeneity is accounted for, the effect of productivity tends to be small or even insignificant. Because columns 3 and 4 of Table 2 include the Mundlak correction, the estimates have to be interpreted as effects of changes in each measure from its time average. In other words, the model is cleaned out from any persistent characteristic, including any high/low level of productivity that is stable over time. Once accounted for this issue, and directly controlled for other structural and strategic determinants, the *variation in firms’ productivity* (on average) is not sufficient to explain the *change* in their *exporting status*. On the one hand, this is because productivity tends to be quite sticky over time. On the other hand, most of the variations of productivity in the short run are caused by the strategies undertaken by the company and its operating environment, which are added as separate regressors in our specification as they represent the drivers of productivity (more on this in Section 5.4). The insignificance of the estimates implies that the remaining component has not enough variation to induce any change in the international attitude. This result can be summarized as follows. While relatively more productive firms tend to show average superior export performance vis-à-vis other companies, the residual role of productivity is blurred when factors laying behind productivity differentials and company level–fixed effects are explicitly accounted for (i.e., controlling for persistent unobserved heterogeneity). In this regard, our estimates show how structural, behavioral, and strategic factors take the center stage as key drivers of international competitiveness in the short run.

This result is largely confirmed by the linear probability models in columns 5 and 6 (especially in the richest specification) that do not impose any restriction on the type of unobserved heterogeneity that takes place. Notice that this finding must not be interpreted as an evidence that productivity does not play any role in firms’ exporting status. The significant effect documented in column 2 suggests a clear positive association between productivity and export. The insignificance portrayed in the following columns indicates that, while there exists a self-selection of persistently more productive companies, there is no effect of changes in productivity on export propensity, once structural and behavioral characteristics of firms are controlled for. Most importantly, the introduction of innovations and the expenditure in R&D keep their significance and prove to be a critical determinant in firms’ internationalization status.^9^

#### Extensive margins: additional tests

There are two main issues that may still affect our results and we have to deal with. The first one has to do with the persistence of the phenomena under consideration, whereby the positive effect of firms’ innovativeness on export may arise from long-lasting internationalization strategies triggering innovative processes and not the other way around. The second interrelated issue has to do with the simultaneity of innovation and export decisions. This empirical strategy will lead to more effectively analyze the relationship between innovation and export competitiveness.

We tackle the first identification challenge by repeating the analysis in Table 2 on the subsample of previously non-exporting companies. Restricting the focus on firms that were not exporters in *t* − 1 is equivalent to modelling firms’ entry on the international markets and it allows us to get rid of any export-driven effects (e.g., introduction of innovations to gain competitiveness in foreign countries). Untabulated results show that innovative strategies keep playing a critical role on the probability of (starting to) export even if the persistent heterogeneity across firms is properly accounted for (see Table A1 in the Online Appendix for details).

To further explore extensive margins, we model innovation and export as simultaneous decisions through a bivariate probit model (once again, augmented with a Mundlak correction for each regressor). Simultaneity may, indeed, generate in-built correlation between the variables if a firm chooses to invest in innovations *only* to penetrate international markets. In this context, bivariate probit models account for this issue by jointly estimating export and innovation via simultaneous equations that perfectly control for (observed and unobserved) third factors that might drive both choices. Results keep showing a positive and significant effect of firms’ innovative strategies on their international propensity, reassuring that our main results do not depend on simultaneity issues. Alternatively, we also implemented SURE regressions with very similar findings (for details and results see the Online Appendix, Table A2a and A2b).

Finally, our estimates are also robust to the inclusion of a rich set of controls for correlated shocks at the region and/or industry levels (through the introduction of industry-specific and province-specific time fixed effects, see Table A3 in the Online Appendix)^10^ and are consistent even we restrict our analysis to the manufacturing sector only.

We run a number of additional exercises to highlight some heterogeneities and sharpen our results.

First of all, we explore differential effects of innovative strategies along the (maximum) geographical extension of the destination market. To this end, we run distinct regressions on a firm’s probability of exporting within the EU area or for exporting beyond the Eurozone. Results confirm the critical role played by firms’ strategy in affecting their exporting status (Table A4 of the Online Appendix). Interestingly, the effect of innovation is found to be significantly more important for farther markets, with a magnitude that is roughly twice as much as the impact for export activity within the Eurozone. On the other hand, the effect of R&D seems to be somewhat reduced. This result might be related to the relatively slow recovery that characterized, during the post-crisis phase, the European domestic market vis-à-vis the Chinese, US, or the UK’s one. In times of fiscal consolidation and austerity at home (Celi et al. 2018), European companies that accessed new foreign markets may have mostly benefited from the increasing demand in USA and China where, in turn, expansionary policies have been in place along the entire post-crisis phase.

An additional exercise aims at exploring heterogeneities along the different types of innovations and separately tests the impact of product, process, and organizational-managerial improvements.^11^ As expected, product innovations dominate other forms of innovativeness (process or organizational-managerial innovations).^12^ This strict dominance is partly related to the fact that new products are the main form of innovation which is not reflected in the level of productivity (which we control for). Moreover, product innovations are linked to technological competitiveness strategies that tend to prevail over other factors capable to explain international performance (see, among others, Dosi et al. 2015). Importantly, product innovations are found to be especially relevant for new exporters, with a magnitude that is roughly twice the impact on the entire sample (4%-increase in the probability to start exporting, as shown in Table A5 in the Online Appendix).

Given the prominent role played by firms’ strategies, we also ask whether, on top of the direct impact of firms’ innovativeness on its international attitude, the upgrading or downgrading paths have additional effects. In other words, two identical companies having the same set of innovative strategies may differ in terms of international propensity depending on *the pattern* undertaken in the recent past (i.e., stable, increasing, or decreasing number of innovative activities). To this end, we enrich the baseline specification with the change in the number of innovative strategies (innovation and R&D) between t − 2 and t − 1. This measure of upgrading takes positive values if the company incremented its array of innovative behaviors, is zero if the firm experienced constant strategies, and takes negative values in case of a reduction in the number of innovative activities (i.e., downgrading). Results clearly show that, on top of the positive direct effect of innovation and R&D, the path of dynamic strategies undertaken in the recent past has a significant impact. In particular, integrating more and more strategies is associated with a positive premium leading to a higher probability of export (ranging between 4 and 8%, as shown in Table A6 of the Online Appendix). On the other hand, the same coefficient suggests that a reduction in the array of dynamic behaviors tends to lower the likelihood of exporting. Notice that these results highlight disproportionate benefits for firms that in the past presented low or reduced innovativeness. For this set of companies, the introduction of one or more dynamic activities would allow to (partially) fill the gap with the group of most dynamic firms.

As a final exercise, we also test the effect of a firm’s innovativeness on its probability of exiting international markets (i.e., fall back on domestic markets). Because of the substantial drop in domestic demand, a large fraction of companies entered the international environment in the aftermath of 2011 (see above). Since a significant flow of firms also exited international markets in the following years, explaining what drives success on international markets is of central importance. We find evidence that companies that withdrew from foreign markets were on average smaller, younger, and, especially, less productive. Notice that while after accounting for unobserved heterogeneity firms’ productivity did not play any role in firms’ entrance, it keeps having a very negative and significant effect on exit strategies. These combined results point at an outflow of companies that attempted to succeed in the international environment despite being less structured and fragile. Importantly, the adoption of R&D and, especially, innovation strategies is found to help overcome structural characteristics by significantly lowering a firm’s probability of exit (−8.5%, as shown in Table A7 of the Online Appendix).

#### Indirect effect on productivity and intensive margins

The analysis presented in the previous sections focused on the direct effect of firms’ innovativeness on export choices. However, on top of this channel, a firm’s innovativeness may also have indirect effects by boosting firms’ long-term productivity which, as we showed in Table 2 and argued in Section 5.1, is conducive of better export performance. Indeed, the complex nature of the innovation-productivity relationship has been at the center of a large set of studies, starting with the seminal contribution of Zvi Griliches (1979). To properly account for the impact that innovation can have on firms’ productivity, at least two elements need to be put at the forefront. First, the delay between technological change, on the one hand, and the possibility of observing an impact in terms of performance, on the other. Innovation needs to settle down, organization needs to adapt, and market effects need to unfold. All this can take time to occur. In addition, it is necessary to take into account the different components of innovation, expliciting the chain of relationship that goes from input to innovative output and, eventually, to companies’ performance (for an extensive empirical exploration of such a chain of relationships, see Crépon et al. 1998).

To explore this additional phenomenon, Table 3 tests the role of innovations on firm productivity growth (RQ2). Columns 1 and 2 present the results of within estimators with firm and time-fixed effects. As expected, higher innovativeness is linked to more pronounced productivity growth (+4% growth rate) that may, in turn, further foster a firm’s probability of export. Interestingly, this indirect effect is largely driven by process and organizational-managerial innovations (column 2), while the introduction of new products is not linked to any productivity growth.

**Table 3:** Indirect effect of innovation on productivity growth

Y:	Productivity growth			
Estimator:	Within estimator		Matching and within estimator	
	(1)	(2)	(3)	(4)
Innovation	0.041**	--	0.233***	--
	(0.019)	--	(0.037)	--
Product inn.	--	0.011	--	0.066
	--	(0.016)	--	(0.049)
Process inn.	--	0.018*	--	0.155***
	--	(0.007)	--	(0.059)
Organiz. inn.	--	0.022**	--	0.133**
	--	(0.007)	--	(0.056)
Constant	−0.117	−0.117	10.476***	10.491***
	(0.101)	(0.101)	(0.019)	(0.019)
Controls				
Region	Yes	Yes	Yes	Yes
Industry	Yes	Yes	Yes	Yes
Time	Yes	Yes	Yes	Yes
Firm FE	Yes	Yes	Yes	Yes
Matching & weights	--	--	Yes	Yes
Observations	13.827	13.827	5.519	5.519
R-squared	0.033	0.034	0.021	0.023

Within estimator with firm and time fixed effects. The dependent variable is the growth rate of productivity (value added per worker). In columns 3 and 4, the analysis is performed after matching techniques (Coarsened Exact Matching) identifying a subsample of companies with the same characteristics (size, age, ex ante productivity, region, sector, and all the other set of controls in Table 1) that only differ for the actual introduction of innovations (the treatment variable). Estimates in columns 3 and 4 are performed employing the matching weights. All measures are defined in the Appendix

*, **, *** denote, respectively, significance at 10%, 5%, and 1% level

Robust standard errors in parentheses

To deal with the possible reverse causality affecting the estimates, we also take advantage of matching techniques in columns 3 and 4. We employ Coarsened Exact Matching to recover a subsample of firms with similar characteristics (size, age, geographical location, industrial features, and, especially, having the same productivity level at the beginning of the sample, 2008) that only differ for the actual introduction of innovations (the treatment variable). We then re-estimate the regressions in columns 1 and 2 on the new (balanced) sample employing matching weights. Once again, firms’ innovativeness is found to have a positive and significant effect on their productivity growth, with a dominant role for process and organizational-managerial innovations (associated to a 15%-higher productivity growth).

Having highlighted the indirect effect of innovation on competitiveness via productivity growth (RQ2), we can now tackle RQ3 concerning the impact of productivity on the intensive margins of export. We capture the latter with the share of sales from exported products (as a percentage of total sales, in columns 1 and 2), or the growth rate of export sales (in columns 3 and 4).

Table 4 relies on within estimators with firm and time-fixed effects and clearly shows that the impact of firms’ innovativeness on export is not limited to the extensive margins, but extends to the performance on the international markets, leading to an 8.3% increase in export growth. Once again, there is a strict dominance of product innovations compared to alternative forms of improvements (columns 2 and 4). Results are qualitatively similar if we employ maximum-likelihood estimator in a selection model a-la Heckman simultaneously taking into account the first-stage decision of whether or not to export (Table A8 in the Online Appendix).

**Table 4:** Intensive margins of export

Y:	Export share		Export sales growth	
	(1)	(2)	(3)	(4)
Productivity	0.450***	0.454***	0.038**	0.038**
	(0.131)	(0.131)	(0.018)	(0.018)
Size	1.255***	1.258***	0.035	0.035
	(0.157)	(0.157)	(0.024)	(0.024)
Age	−0.095	−0.098	−0.049***	−0.049***
	(0.102)	(0.102)	(0.014)	(0.014)
R&D share	0.107***	0.102***	0.007***	0.007***
	(0.016)	(0.016)	(0.001)	(0.001)
Innovation	0.584***	--	0.083***	--
	(0.187)	--	(0.019)	--
Product inn.	--	0.956***	--	0.061***
	--	(0.235)	--	(0.023)
Process inn.	--	0.028	--	0.012
	--	(0.262)	--	(0.026)
Organiz. inn.	--	0.408*	--	0.040*
	--	(0.223)	--	(0.023)
Export sales	--	--	−0.434***	−0.434***
	--	--	(0.013)	(0.013)
Constant	4.701***	4.541**	7.213***	7.211***
	(0.177)	(0.177)	(0.571)	(0.572)
Controls				
Region	Yes	Yes	Yes	Yes
Industry	Yes	Yes	Yes	Yes
Time	Yes	Yes	Yes	Yes
Region*Time	Yes	Yes	Yes	Yes
Industry*Time	Yes	Yes	Yes	Yes
Firm FE	Yes	Yes	Yes	Yes
Observations	67.108	67.108	16.954	16.954
R2	0.008	0.009	0.184	0.184

Within estimators with firm and time fixed effects. The dependent variable is the intensive margin of export defined as the share of exported sales on total turnover (Export share in columns 1 and 2) or as the growth rate of exported value (Export sales growth, in columns 3 and 4). Additional regressors (untabulated) follow the specification in Table 1. All measures are defined in the Appendix

*, **, *** denote, respectively, significance at 10%, 5%, and 1% level

Robust standard errors in parentheses

As an additional exercise, we also exploited quantile regressions to emphasize non-linearities in the effect of firms’ innovativeness on export shares (Table A9 in the Online Appendix). Results highlight that research activities are fundamental to improve the performance on foreign markets except in case of firms that are extremely large exporters.^13^ In a similar vein, the introduction of innovations is a relevant strategy especially for marginal exporters while playing a minor role for companies that rely heavily on export. Taken together, this result points to the critical role of R&D and innovation in penetrating foreign markets. However, once the firm reaches a significant degree of dependence from the international environment, its degree of innovativeness has a reduced impact while size and productivity stand out as key elements for competitive advantages. Importantly, if we repeat the analysis on the overall firm performance (i.e., total sales growth), results show a significantly reduced (albeit positive) role for R&D and innovative activities, likely underlying the existence of static low-productive firms operating domestic market niches whose performance is not affected by any form of dynamic strategy (Table A10 in the Online Appendix).

### Micro-sized and less productive firms

Once the effect of dynamic strategies on firms’ international propensity is established, we now turn the attention to RQ4, concerning potential non-linearities in the effects of interest. In particular, smaller and less productive firms may have disproportionate benefits from the introduction of innovations and the investment in R&D projects. To test for this heterogeneity, Table 5 interacts the effect of Innovation and R&D with dummy indicators for higher and lower productivity or larger and smaller size. The thresholds used to identify fragile companies are listed in the third rows (33rd or 25th percentile of the 2008 cross-sectional distribution of size and productivity). Notice that, although our results all relate to SMEs because of the composition of our dataset, this exercise allows to highlight the additional role of dynamic strategies for truly nano-sized companies, as the thresholds for smaller firms identify units with less than six and four employees, respectively.

**Table 5:** Disproportionate effects for small or less productive firms

Y:	Export				Export sales growth			
Estimator:	RE-Probit with Mundlak		RE-Probit with Mundlak		Within estimator		Within estimator	
Fragility measure:	Size		Productivity		Size		Productivity	
Threshold fragile:	33rd	25th	33rd	25th	33rd	25th	33rd	25th
	(1)	(2)	(3)	(4)	(5)	(6)	(7)	(8)
Innovation*Sound	0.0124***	0.011***	0.017***	0.016***	0.078***	0.089***	0.071***	0.080***
	(0.003)	(0.003)	(0.003)	(0.003)	(0.020)	(0.019)	(0.020)	(0.020)
R&D share*Sound	0.002***	0.002***	0.002***	0.002***	0.007***	0.007***	0.007***	0.007***
	(0.000)	(0.000)	(0.000)	(0.000)	(0.002)	(0.002)	(0.002)	(0.002)
Innovation*Fragile	0.0442***	0.0481***	0.053***	0.051***	0.219***	0.141**	0.175***	0.161***
	(0.003)	(0.003)	(0.003)	(0.003)	(0.049)	(0.068)	(0.035)	(0.041)
R&D share*Fragile	0.003***	0.003***	0.004***	0.004***	0.024***	0.030***	0.012***	0.011***
	(0.000)	(0.000)	(0.000)	(0.000)	(0.007)	(0.011)	(0.004)	(0.004)
Constant	−3.115***	−3.110***	−3.164***	−3.174***	7.017***	7.002***	6.992***	6.968***
	(0.212)	(0.212)	(0.240)	(0.240)	(0.588)	(0.588)	(0.588)	(0.588)
Controls								
Region	Yes	Yes	Yes	Yes	Yes	Yes	Yes	Yes
Industry	Yes	Yes	Yes	Yes	Yes	Yes	Yes	Yes
Time	Yes	Yes	Yes	Yes	Yes	Yes	Yes	Yes
Region*Time	Yes	Yes	Yes	Yes	Yes	Yes	Yes	Yes
Industry*Time	Yes	Yes	Yes	Yes	Yes	Yes	Yes	Yes
Mundlak	Yes	Yes	Yes	Yes	--	--	--	--
Firm FE	--	--	--	--	Yes	Yes	Yes	Yes
Observations	45.401	45.401	37.71	37.71	16.424	16.424	16.424	16.424
Pseudo R2	0.759	0.759	0.767	0.767	--	--	--	--
R2	--	--	--	--	0.206	0.205	0.206	0.205

RE-probit models with Mundlak correction (marginal effects in columns 1–4) and within estimator with firm and time fixed effects (in columns 5–8). The dependent variable is the extensive margin of export (Export), a dummy variable identifying exporting companies in columns 1–4, or the growth rate of exported value (Export sales growth in columns 5–8). Fragile and Sound are dummy measures identifying firms whose Size (number of employees) or Productivity (log value added per worker) at the beginning of the sample is respectively below or above the 33rd or 25th percentile (threshold in the third row) of the respective cross-sectional distribution

All measures are defined in the Appendix

*, **, *** denote, respectively, significance at 10%, 5%, and 1% level

Robust standard errors in parentheses

Both the extensive (columns 1–4) and the intensive (columns 5–8) margins of export present significant non-linearities pointing to larger benefits for smaller and less productive companies (for instance, innovations of less productive companies are linked to a 5.3% increase in the probability of export and 17% higher export sales growth, which are reduced to 1.7% and 7.1% in case of innovations undertaken by a productive firm). This evidence is somewhat consistent with the recent analysis of Hernández (2020). This is a critical finding as it identifies innovative strategies as a potential tool to fill the gap between large/productive companies and the set of less structured firms that are ideal targets for policy measures.

## Concluding remarks

Overall, we contribute to the debate on Italian external competitiveness by pointing out the importance of behavioral and strategic factors for SMEs in shaping firm-level competitive advantages. While our results confirm the positive effect of structural characteristics, such as size and productivity, they also highlight the existence of additional factors that help boost firms’ internationalization. Among these, innovation and R&D play a crucial role and affect external competitiveness both directly and via productivity improvements. Even though the positive effect of innovative activities and R&D is certainly not new, we explore several new levels of heterogeneity, as well as indirect effects that may provide a sound base for the construction of granular policy implications. In particular, the analysis allows for the identification of some key characteristics of firms that favor their external competitiveness in a phase of deep economic crisis.

First, we showed that while firms exhibiting a higher productivity do perform better, *changes* in productivity are not significantly associated to improvements in external competitiveness, once other firm-level structural and behavioral characteristics, including innovation strategies, are controlled for. In other words, *temporary changes in productivity* do not have an impact on export performance, while long-term, persisting productivity premia do. This might signal that it is only when innovation and R&D become less sporadic (more systematic) that behaviors translate into structural and persisting advantages (e.g., via the accumulation of complementary competencies, routines, and resources) and determine *long-term shifts in productivity*. It is these shifts in long-term productivity, and not temporary changes, that eventually yields improvements in external competitiveness.

Second, firms expanding the *array of product*, *process*, and *organizational innovations* significantly improve their capacity to penetrate foreign markets. In a similar vein, the adoption of innovative strategies is found to lower the probability of exiting foreign markets. By documenting these facts, together with the indirect impact of innovative activities—especially process and managerial innovation—on long-term productivity, we provide sound evidence on the importance of favoring technological and organizational change as a source of external competitiveness, particularly in a phase of crisis.

Third, *most fragile firms*, including the least productive and smaller companies, are the ones that appear to gain the greatest benefits from innovation strategies in terms of external competitiveness, especially in terms of export growth. In troubled times, as the ones that we are living in the aftermath of the big financial crisis worsened by the effects of COVID-19 outbreak, our results provide a strong argument in favor of comprehensive recovery strategies based on multiple strategies and multiple actors.

In times of dramatic crises there seems to be *no easy recipe* when it comes to public policies. On the one hand, our study highlights that “cherry picking” is not likely to be the way out of deep economic downturns. In fact, it appears that SMEs are not only the most resilient firm category (often acting as potential stabilizers during crises and in their immediate aftermath); small and least productive firms are also likely to benefit the most from the adoption of innovation strategies. Hence, helping weak cherry trees to grow stronger might turn out to be better than strengthening cherry trees that are already robust. On the other hand, SMEs and particularly those exhibiting the lowest productivity are also the least capable to undertake innovation efforts, in spite of the benefits they could obtain from these strategies. This is consistent with the abundant literature showing that, while more flexible and resilient, SMEs are also less able to adopt up-to-date technology and new organizational devices, and may not be able to fully recover from downturn periods. Therefore, there is urgent need for careful innovation policies targeted towards SMEs. Our work suggests that subsidizing R&D is only a minor part of the story. This is not only because SMEs are generally not prone to set up R&D labs and to undertake formal research and innovation activities. In fact, it appears that an effective response to crises implies the adoption of a wide variety of innovation strategies, combining technical change with product and organizational innovation. Hence, policies should favor a broad range of training and investment activities leading to a generalized reduction of barriers to technical and product innovation, but also, and most significant, to more effective organizational structures and capabilities. Policies oriented to favour substantial organizational innovation include ambitious measures aimed at helping SMEs to grow multinational, which would yield inter alia a significant increase in resilience to downturns and in external competiveness, as our study has shown.

More research is needed to develop a deeper understanding of factors and mechanisms underlying resilience to big crises, and of policies to strengthen the capabilities of firms to anticipate, tackle, and respond to downturns. In this respect, our work has clear limitations that need be overcome in future research. First, the characteristics of successful innovation as well as structural and strategic strengths and weaknesses of firms cannot be captured in details by means of the available data. This is most likely to require a combination of quantitative and qualitative analyses based on case studies. Second, given the main focus of our study on firm level determinants, we have deliberately disregarded the sectoral patterns of external competitiveness. Future research would definitely benefit from examining how industry-level characteristics affect, and interact with, firm-level factors and strategies. Third, the involvement of SMEs in global value chains needs to be taken into account in order to better evaluate their degree of exposure to economic downturns as well as their capabilities to flexibly react to crises. This might represent a key factor in examining other times of crisis, such as the ongoing pandemic developments. There is ample room for additional future research in this regard.

## Supplementary Information

ESM 1 (DOCX 73 kb)

## Appendix

**Table 6:** Variable definition

Variable	Definition
Export	Dummy variable for exporting companies
Export share	Share of sales from exported products
Export sales	Log-value of exported products (lagged)
Export sales growth	Δln(export sales)
Import	Dummy variable for importers
Productivity	ln(value added / # employees)
Size	ln(# employees)
Age	ln(1 + age)
Capitalisation	ln(physical capital / # employees)
Group	Dummy variable for group membership
Innovation	Dummy variable for the introduction of innovations (independently of the type)
Product inn.	Dummy variable for the introduction of product innovations
Process inn.	Dummy variable for the introduction of process innovations
Organiz. inn.	Dummy variable for the introduction of organizational-managerial innovations
R&D share	Expenditure in R&D (as a share of sales)
Sales	Sales / total assets
Sales growth	Δln(sales)
ROA	Net income / total assets
Vertical integration	Value added / total turnover
Cost of labour	ln(Cost of labour / total cost of production)
Leverage	Total assets / equity
Net acc. payable	(Accounts payable - accounts receivable) / total assets
Bank debt	Bank debt / total assets
Human capital	% of graduated employees (tertiary education)
Domestic network	Dummy variable for local domestic relationships with other companies

## Footnotes

1 With specific reference to the Italian case, Storm (2019) highlighted how the contraction of domestic demand contributed to prolong and exacerbate the impact of the 2008 crisis.

2 See, among others, De Loecker (2007), Lileeva and Trefler (2010), Bustos (2011), Mayer et al. (2014, 2016).

3 In an earlier contribution, Santarelli and Sterlacchini (1990) point out that when SMEs innovate, they often do so lacking adequate financial and managerial resources and relying on informal procedures. As a result, SMEs tend to perform a significant amount of their innovative activities in areas such as design, production, and marketing rather than in R&D (which often do not exist at all).

4 Overall, SMEs are less likely to perform large and formalized R&D projects. As a result, relying on R&D expenditure as a way to measure SMEs’ innovative performance may result in an incomplete description of the actual innovative effort. However, the relative importance of R&D efforts within the overall SMEs’ innovative strategy may vary significantly according to the sectoral specialization.

5 A parallel strand of the Italian literature focused on the effect on external competitiveness of resource misallocation (Calligaris et al. 2018), ownership characteristics (Corbetta 1995; Montemerlo 2005; Cerrato and Piva 2012; Bianco et al. 2013), and managers’ education (Cucculelli and Micucci 2008).

6 Additional details are provided in the Online Appendix.

7 More details on the definition of all the variables employed can be found in Appendix.

8 The importance of belonging to multinational groups as drivers of expert capabilities is consistent with the insight put forth by Dunning (1993:79-86) that multinational status is associated with “economies of common governance” stemming from their experience of foreign markets yielding greater organizational skills and capabilities to exploit foreign markets.

9 Notice that the (apparently small) coefficient of R&D has to be interpreted as the effect of a 1% increase in the expenditure in R&D. For instance, a firm increasing R&D expenditure up to 10% of its total sales has a 3% higher probability of exporting.

10 This allows to capture any shock that is time varying and common to firms within a specific geographical area or industrial sector. This may be particularly relevant in the aftermath of the Great Recession in which demand shocks may be not homogeneous and hit more severely specific segments of the market.

11 Coherently with the Community Innovation Survey (CIS) organizational-managerial innovations comprehend new organizational methods in a firm’s business practices (including knowledge management), workplace organization, or external relations that has not been previously used.

12 We also allowed for combined effects, interacting product and process innovations with organizational forms of improvements. Results show no significant interaction terms, pointing at an impact that is additive rather than multiplicative.

13 This is because highly internationalized firms typically lean on alternative strategies to gain/preserve the competitive advantage over their rivals (e.g., outsourcing, market power exploitation, strict connection with foreign markets).
