# Supplementary material for: Innovation drivers of external competitiveness in the great recession
Source: Small Bus Econ (Dordr). 2021 Jan 29;58(3):1497–516. doi: 10.1007/s11187-021-00453-0 (PMC7846269; doi:10.1007/s11187-021-00453-0)
Supplement: Supplementary file 1 — (DOCX 73 kb) [file 11187_2021_453_MOESM1_ESM.docx]

Innovation Drivers of External Competitiveness in the Great Recession

**Online Appendix**

# THE MET SURVEY

The MET survey is the widest survey administrated in a single European country and it is specifically conceived to study Italian firms’ characteristics and strategies, with particular attention to their internationalization process, innovative behaviour, and network relationships. Its representativeness is based on a sample design that is stratified along three dimensions: size class, sector, and geographical region.

MET survey’s population of interest refers to the enterprises belonging to all the size classes operating within Industry (construction excluded) and Production Services sectors (overall 38 NACE Rev.2 3 digit sectors), accounting for 60% and 40% of the sample, respectively. ^[[1]](#footnote-1)^

The employed calibration estimators allow the survey, under predetermined circumstances, to reproduce known population parameters as well as to recover possible sample biases following from the (non-random) missing responses’ distribution. The computation of the calibration estimators takes also advantage of a set of auxiliary information (other than the one employed to identify the strata) drawn from the population of interest. This auxiliary information constitutes a further binding constraint the final sample has to reproduce. All the constraints are drawn from ISTAT Italian Statistical Business Register (ASIA) while the calibration procedure makes use of an ad-hoc iterative algorithm. As for the longitudinal part of the sample, a specific calibration estimator has been computed to account for firms interviewed in two succeeding waves.

**Composition of the MET database by firms’ size class.**

|  | **2008** | **2009** | **2011** | **2013** | **2015** |
| --- | --- | --- | --- | --- | --- |
| Micro (1-9) | 38.4% | 60.0% | 61.6% | 48.1% | 46.9% |
| Small (10-49) | 38.4% | 26.0% | 24.7% | 33.6% | 34.6% |
| Medium (50-249) | 19.5% | 10.5% | 10.6% | 13.5% | 14.7% |
| Large (>250) | 3.00% | 3.50% | 3.10% | 4.80% | 3.70% |
| # observations | 24,896 | 22,340 | 25,090 | 25,000 | 23,071 |

On top of that, starting from the wave 2009 the sampling scheme employs Bayesian techniques in order to ensure high precision estimates on a selected range of topics that represent the core part of the survey (R&D, innovation and internationalization strategies among others). These techniques draw on a tree-based classification model able to detect, in the preceding wave, the strata showing higher rates of enterprises undertaking these relevant activities. This procedure has required further constraints leading to an oversampling of the targeted strata. Clearly, such sample bias is accounted for during the calibration estimator’s procedure and is perfectly controlled for in the econometric analysis.

Before moving to additional econometric details and to the extensive set of robustness checks, it is worth providing full details on the exact questions underlying to the measures employed in the analysis, and how such measures are computed.

A 1 “In the last years, did your firm perform economic activities abroad?” (Yes/No)

If yes:

2 “In what form?” (Direct Export/Indirect Export/Import/Joint venture/….) (Yes/No)

Export: dummy =1 if A1=Yes *and* (A2= Direct Export *or* A2= Indirect Export), 0 otherwise.

B “In what market(s) does your firm sell its product/services? Provide a breakdown by geographical area.”

B1. In the same local area of the firm (%).

B2. In the same region of the firm (%).

B3. In other Italian regions (%).

B4. In other countries within the European Union (%).

B5. In other countries outside the European Union (%).

Export share: B4+B5.

Export sales: Export share * Total sales (from balance sheets).

Export sales growth: log-change in Export sales.

C “In the last years, did your firm introduce the following innovations?”

C1. Radical product innovations (Yes/No).

C2. Marginal product innovations (Yes/No).

C3. Radical process innovations (Yes/No).

C4. Marginal process innovations (Yes/No).

C5. Organizational, managerial, or commercial innovations (Yes/No).

Notes in the questionnaire: Radical Product innovations involve the introduction of a totally-new product or service (or a substantial restyling). Marginal Product innovations involve the introduction of a significantly-improved product or service. Radical Process innovations entail a significant change in the production process (e.g., development and introduction of a new production process). Marginal Process innovations are related to a marginal change in the production process.

Innovation: dummy =1 if (C1=Yes or C2=Yes or C3=Yes or C4=Yes or C5=Yes), 0 otherwise.

Product inn: dummy =1 if (C1=Yes or C2=Yes), 0 otherwise.

Process inn: dummy =1 if (C3=Yes or C3=Yes), 0 otherwise.

Organiz. inn: dummy =1 if C5=Yes, 0 otherwise.

D “In the last years, did your firm perform activity of Research and Development?” (Yes/No)

If yes:

D1. “What is the overall expenditure in R&D as a share of firms’ turnover?”

D2. “What was the share of R&D that is performed in-house?”

R&D: dummy =1 if D=Yes, 0 otherwise.

R&D share: D1 (if D=Yes), 0 otherwise.

E “Does your firm belong to a corporate group?” (Yes/No)

If yes:

E1. “What is the nationality of the group?” (Italian/EU/Extra-EU)

Group: dummy =1 if E=Yes, 0 otherwise.

Group multinational: dummy =1 if E=Yes & (E1=EU or E1=Extra-EU), 0 otherwise.

F “In the last years, did your firm invest in tangible or intangible assets?” (Yes/No)

Investment: dummy =1 if F=Yes, 0 otherwise.

G1. “How many employees does your firm have?”

G2. “In what year did your firm start its activity?”

Size: ln(1+G1).

Age: ln(1+T-G2), where T is the year of the survey.

# ECONOMETRIC STRATEGY ADDENDUM

Dealing with unobserved heterogeneity in a binary-response framework is not trivial. On the one hand, standard random-effects (RE) models impose unrealistic assumptions on the type of heterogeneity that takes place (*i.e.*, $c_{i}$ must be uncorrelated with the entire set of regressors). On the other, fixed-effects models, that do not impose any hypothesis on $c_{i}$, are computationally difficult and introduce an incidental parameter problem leading to inconsistent estimates. Our strategy is in-between the two approaches and relies on RE-probit models augmented with the time average of each regressor (*i.e.*, Mundlak-type controls).

The standard RE-probit model on the full set of covariates

$\Pr\left( Y_{it}=1|Z_{it-1},c_{i} \right)=\Phi(\beta^{'}Z_{it-1}+c_{i}+\varepsilon_{it})$ ( 2 )

imposes $c_{i}|Z_{i}\sim N\left( 0,\sigma_{c}^{2} \right)$, which implies the assumption of strict independence between firm-specific unobserved heterogeneity ($c_{i}$) and the full set of regressors. Because this unrealistic hypothesis would generate biased results in our setup, we follow Wooldridge (2010) and relax it by focusing on the effect of each variable in terms of deviations from its time average. This allows us to purge the model from persistent heterogeneity across firms and to derive cleaner policy implications. Our estimator can be viewed as a Mundlak (1978) version of the Chamberlain (1980)’s assumption on the correlation between $c_{i}$ and Z, which requires the milder hypothesis of: $c_{i}|Z_{i}\sim N\left( \psi+\theta^{'}\bar{Z}_{i},\sigma_{a}^{2} \right)$, where $\sigma_{a}^{2}$ is the variance of $a_{i}$ in $c_{i}=\psi+\theta^{'}\bar{Z}_{i}+a_{i}$, and $\bar{Z}_{i}$ is the time-average of $Z_{it-1}$ (see Brancati et al., 2017). Notice that this approach is equivalent to a fixed-effects model in which the heterogeneity is projected on the time-mean of the regressors ($\bar{Z}_{i}$), allowing to write the latent variable as $Y_{it}^{*}=\psi+{\beta^{'}Z_{it-1}+\theta}^{'}\bar{Z}_{i}+a_{i}+e_{it}$, with $e_{it}\sim N\left( 0,1 \right)$. As usual, the estimator hinges on the exogeneity of $Z_{it-1}$ conditional on $c_{i}$. We verify this hypothesis by adding the vector $Z_{it}$ to our specification and testing the significance of its estimates (as proposed by Wooldridge, 2010). The test never rejects the null, thus providing at least some justification for the strict exogeneity assumption.

# SIMULTANEITY OF INNOVATION AND EXPORT

One important issue that, for the sake of brevity, is overlooked in the paper has to do with the simultaneity of the innovation and export decisions. While we always match current export activity with lagged innovative choices, there is still the possibility of significant correlation between the two equations that may invalidate the coefficients of interest. For instance, firms’ may choose to invest in innovations to penetrate international markets. If this is the case, the two choices are simultaneously determined and this issue should be properly accounted for. The second point has to deal with the identification of the determinants of firms’ innovative strategies, which are especially useful to provide some policy guidance.

To this aim, we account for the simultaneity of the phenomena by employing bivariate probit models (with Mundlak correction). The specification estimates firms' probability of exporting conditionally on its (lagged) innovative status. The model can be summarized by the following system of equations:

$$\left\{ \begin{aligned} Export_{it}=1 if Export_{it}^{*}={\theta Innovation_{it-1}+\beta}_{1}^{T}X_{1it-1}+\varepsilon_{1it}>0 \\ Export_{it}=0 if Export_{it}^{*}=\theta Innovation_{it-1}+\beta_{1}^{T}X_{1it-1}+\varepsilon_{1it}\leq0 \end{aligned} \right.$$

$$\left\{ \begin{aligned} Innovation_{it-1}=1 if Innovation_{it-1}^{*}=\beta_{2}^{T}X_{2it-2}+\varepsilon_{2it-1}>0 \\ Innovation_{it-1}=0 if Innovation_{it-1}^{*}=\beta_{2}^{T}X_{2it-2}+\varepsilon_{2it-1}\leq0 \end{aligned} \right.$$

where $Export_{it}$ and $Innovation_{it-1}$ are the observed (dummy) dependent variables, $Export_{it}^{*}$ and $Innovation_{it-1}^{*}$are latent variables, while $\varepsilon_{1it}$ and $\varepsilon_{2it-1}$ are the two error terms, assumed to be *i.i.d.* as a bivariate normal with unitary variance and correlation coefficient $\rho=corr\left( \varepsilon_{1},\varepsilon_{2} \right).$ The system of equations allows to estimate the impact of innovativeness on export, and to jointly analyze the drivers of innovative strategies. $X_{1it-1}$ is the standard set of regressors in Table 1, while $X_{2it-2}$ is a vector of determinants for innovations, including R&D choices (internal vs outsourcing), structural characteristics (size, age, capitalization, and productivity), proxies for demand conditions (past sales growth), operating environment (group belonging and participation in local networks), as well as measures aimed at capturing firms’ financial conditions.

Table A3a presents the main results. First, even after accounting for the simultaneity of the phenomena, and perfectly controlling for third factors that jointly affect export and innovation, firms’ innovative strategies are found to have strong and significant effects on their international propensity. In other words, the main results of the paper are found to be robust and not to depend on simultaneity issues.

The analysis on the determinants of firms’ innovativeness present interesting results. As expected, structural characteristics are significantly affecting the introduction of innovations, especially in case of larger, younger, and more productive companies. Similarly, past sales growth, which can be thought of as a proxy for demand trends, is positively associated to firms’ innovativeness. The existence of R&D projects is clearly correlated to huge increases in the probability of introducing innovations. Importantly, this effect is not limited to the investment in R&D performed within the firm (internal R&D), but extends to firms outsourcing R&D activities, even though with smaller magnitudes (+8% vs. +27% probability). This is a relevant result, as the outsourcing of R&D projects is widely employed by very small firms that are not structured enough to undertake internal research projects.^[[2]](#footnote-2)^ Another important factor in driving firms’ innovativeness is related to the operating environment of a firm. Belonging to a corporate group significantly increases the probability of innovating by roughly 5%, as well as the insertion into domestic networks with other companies (4.5%). This result is largely in line with the dominant literature on domestic districts, whereby close proximity with other companies may foster the innovative process through iterated exchanges of knowledge flows. Notice that, once again, the insignificance of Human capital may be driven by its persistence (we always account for Mundlak correction) as well as controls for R&D capturing most of its effect.

Finally, columns 1 and 2 emphasize the critical role played by financial constraints in the development of innovative projects. By their very own nature, innovative firms, especially SMEs, are more likely to suffer from financial problems. Because of their informational opaqueness, their little tangible assets to pledge as collateral, and the riskiness of their strategies, most potentially-innovative firms are credit-rationed and face relevant obstacles in financing their investments. In this regard, the type of innovation to be financed, the characteristics of the firm, and its relationship with the lender bank play a crucial role in the actual capability of introducing innovations.

We analyze this effect in two alternative ways. In column 1, we employ a synthetic measure of firms’ creditworthiness (or bankability), defined as the first principal component (Creditworthiness) of several financial rations that are traditionally used by banks to compute internal credit scores (leverage, ability to pledge collateral, age, size, cash flow to total assets ratio, sales to total assets ratio, proxies for rollover risk, …).^[[3]](#footnote-3)^ As expected, this measure is positively associated to firms’ innovativeness, possibly because of its correlation with firm access to external credit.

As an alternative measure, column 2 employs the predicted probability of being financially constrained. In particular, the MET survey contains information on the existence of positive net-present-value investment projects that were not undertaken because of a lack of financial means. This measure can be thought of as a direct proxy for financial constraints (dummy variable) and is employed in Table A3b as a dependent variable.

On the top of some structural characteristics, we employ a set of measures that are traditionally used by the literature as proxies for relationship lending. Relationship lending represents the informational privilege that a bank accumulates over time by establishing close ties with its borrower so to overcome problems of informational asymmetry, especially when dealing with innovative projects. The amount of soft information gathered by banks can be critical in determining credit access, especially for opaque SMEs whose hard information is not enough to correctly evaluate firm creditworthiness. In other words, the existence of close ties with the lender bank significantly helps overcoming credit constraints.

Given the unobservability of the stock of prior information accumulated by the bank, we employ three main measures to capture this phenomenon. The first one, Bank distance, is the physical distance between the belonging province of a company and the headquarter of the lending bank (in log-Km). This measure is correlated with the “informational distance” between the lender and the borrower; the larger the distance, the higher banks’ difficulties in transmitting soft information to the headquarters. Similarly, banks' degree of hierarchization (here proxied by Bank size, i.e., the log of banks’ total assets) significantly affects the transmission of soft information gathered from delocalized branches to the upper levels. Finally, the higher the number of banks each firm is borrowing from, the weaker the relationship that is established with the lender, and the lower the amount of soft information that is allegedly gathered.

Table A3b presents results that are in line with a priori expectations and emphasize the critical role played by relationship lending in reducing a firm’s probability of being financially constrained. Finally, in column 2 of Table A3a we employ the predicted probability from Table A3b to show the detrimental effect of financial constraints on firms’ innovativeness, which is always very negative and extremely significant, suggesting that finance may represent a severe obstacle for financing innovative activities of SMEs. Our results are broadly consistent if we employ alternative techniques to deal with simultaneity issues, such as Seemingly Unrelated Regression (SURE) models in columns 3 and 4.

# ADDITIONAL TABLES AND ROBUSTNESS

**Table A1: Extensive margins of export: previously non-exporting companies**

| Y: | Export | | | |
| --- | --- | --- | --- | --- |
|  | (1) | (2) | (3) | (4) |
| Innovation | 0.022*** | 0.022*** | 0.023*** | 0.019*** |
|  | (0.007) | (0.006) | (0.007) | (0.006) |
| R&D share | 0.002*** | 0.002*** | 0.002*** | 0.003*** |
|  | (0.001) | (0.001) | (0.001) | (0.001) |
| Constant | -1.806*** | -0.197*** | -1.451*** | -0.171*** |
|  | (0.323) | (0.052) | (0.422) | (0.053) |
| Controls |  |  |  |  |
| Region | yes | yes | yes | yes |
| Industry | yes | yes | yes | yes |
| Time | yes | yes | yes | yes |
| Region*Time | no | no | yes | yes |
| Industry*Time | no | no | yes | yes |
| Mundlak | yes | -- | yes | -- |
| Firm FE | -- | yes | -- | yes |
| Observations | 11.599 | 32.889 | 11.599 | 30.54 |
| Pseudo R2 | 0.554 | -- | 0.582 | -- |
| R2 | -- | 0.124 | -- | 0.164 |

*Notes: RE-probit models with Mundlak correction (marginal effects in columns 1-2) and linear probability models with firm and time fixed effects (columns 3-4). The sample is restricted to companies that were not exporting in* $t-1$*. Untabulated controls follow the specification in Table 1. The dependent variable is the extensive margin of export (Export). All measures are defined in Appendix. *, **, *** denote, respectively, significance at 10%, 5%, and 1% level. Robust standard errors in parentheses.*

**Table A2a: accounting for simultaneity in export and innovation decisions**

|  |  |  |  |  |
| --- | --- | --- | --- | --- |
|  | (1) | (2) | (3) | (4) |
| **Export equation:** | | |  |  |
| Innovation | 0.493*** | 0.504*** | 0.082*** | 0.076*** |
|  | (0.020) | (0.019) | (0.015) | (0.011) |
| R&D | 0.003*** | 0.002*** | 0.004*** | 0.003*** |
|  | (0.000) | (0.000) | (0.001) | (0.001) |
| Investment | 0.029*** | 0.022*** | 0.026* | 0.028* |
|  | (0.007) | (0.007) | (0.014) | (0.014) |
| **Innovation equation:** | | |  |  |
| External R&D | 0.061*** | 0.082*** | 0.070*** | 0.066*** |
|  | (0.011) | (0.012) | (0.011) | (0.011) |
| Internal R&D | 0.247*** | 0.273*** | 0.104*** | 0.122*** |
|  | (0.009) | (0.010) | (0.010) | (0.009) |
| Creditworthiness | 0.016*** | -- | 0.011*** | -- |
|  | (0.005) | -- | (0.001) | -- |
| Prob (financial constraints) | -- | -0.419*** | -- | -0.122* |
|  | -- | (0.167) | -- | (0.072) |
| Human capital | 0.008 | 0.023 | 0.005 | 0.005 |
|  | (0.017) | (0.017) | (0.015) | (0.015) |
| Size | 0.027*** | 0.027*** | 0.052*** | 0.052*** |
|  | (0.003) | (0.003) | (0.007) | (0.007) |
| Productivity | 0.013*** | 0.0115*** | 0.012*** | 0.012*** |
|  | (0.003) | (0.003) | (0.001) | (0.001) |
| Capitalisation | -0.001 | 0.001 | 0.001 | 0.001 |
|  | (0.001) | (0.001) | (0.001) | (0.001) |
| Age | -0.008** | -0.0259*** | 0.005 | 0.005 |
|  | (0.003) | (0.005) | (0.011) | (0.011) |
| Sales growth | 0.018** | 0.021** | 0.021*** | 0.021*** |
|  | (0.008) | (0.008) | (0.008) | (0.008) |
| Local network | 0.045*** | 0.040*** | 0.032*** | 0.032*** |
|  | (0.006) | (0.006) | (0.007) | (0.007) |
| Group | 0.050*** | 0.044*** | 0.033*** | 0.033*** |
|  | (0.008) | (0.009) | (0.006) | (0.006) |
| Model | Bivariate probit | | SURE | |
| Controls |  |  |  |  |
| Region | yes | yes | yes | yes |
| Industry | yes | yes | yes | yes |
| Time | yes | yes | yes | yes |
| Mundlak | yes | yes | yes | yes |
| Observations | 20.684 | 20.684 | 20.684 | 20.684 |
| Rho | -0.641*** | -0.641*** | -- | -- |

*Notes: bivariate probit models with Mundlak correction (marginal effects) and Seemingly Unrelated Regression (SURE) estimates (in columns 1-2 and 3-4, respectively). The top panel reports estimates from the export equation (untabulated regressors follow the specification in Table 1). The bottom panel presents the innovation equation. External and Internal R&D are dummy variables identifying companies outsourcing R&D or performing R&D internally. Creditworthiness is a synthetic proxy for firms’ creditworthiness computed as the first principal component of several financial characteristics that may affect a bank’s decision to lend (leverage, collateral availability, size, rollover risk, age). Prob(financial constraints) is the predicted probability of being financially constrained as computed in the following table (D.8.B). Rho is the estimated correlation of the error terms of the two equations. All measures are defined in Appendix. *, **, *** denote, respectively, significance at 10%, 5%, and 1% level. Robust standard errors in parentheses.*

**Table A2b: estimation of predicted probability of financial constraints**

| Y: | Financial Constraints |
| --- | --- |
|  | (1) |
| Age | -0.032*** |
|  | (0.001) |
| Size | -0.015*** |
|  | (0.002) |
| Bank distance | 0.0104*** |
|  | (0.000) |
| Bank size | 0.002** |
|  | (0.001) |
| N banking relationships | 0.003*** |
|  | (0.001) |
| Observations | 73.865 |
| Pseudo R2 | 0.122 |

*Notes: Probit model (marginal effects, in units of standard deviations in columns 1-2). The dependent variable is a direct measure of financial constraints (self reported) identifying firms having positive net-present-value projects that were not undertaken because of a lack of financial resources. Bank distance is the physical distance (log-Km) between the belonging province of a firm and the headquarter of its lending bank (in case of multiple banking relationships, an average has been applied). Bank size is the log of total assets of the lending bank. N banking relationships is the number of banking relationships in place. All measures are defined in Appendix. *, **, *** denote, respectively, significance at 10%, 5%, and 1% level. Robust standard errors in parentheses.*

**Table A3. Extensive margins of export: controlling for interacted time effects**

| Y: | Export | | | |
| --- | --- | --- | --- | --- |
| Estimator: | RE-Probit with Mundlak | | Linear probability model | |
|  | (1) | (2) | (3) | (4) |
| Productivity | -0.0002 | 0.002 | 0.004 | 0.006 |
|  | (0.0017) | (0.002) | (0.004) | (0.011) |
| Size | 0.0177*** | 0.0317*** | 0.020*** | 0.050*** |
|  | (0.0032) | (0.0044) | (0.004) | (0.012) |
| Age | 0.000 | -0.001 | 0.000 | -0.005 |
|  | (0.002) | (0.002) | (0.003) | (0.006) |
| Capitalisation | 0.0012 | 0.002 | 0.000 | 0.005 |
|  | (0.0013) | (0.002) | (0.001) | (0.004) |
| Group | 0.0127* | 0.022*** | 0.028*** | 0.046*** |
|  | (0.0068) | (0.008) | (0.008) | (0.015) |
| Import | 0.084*** | 0.074*** | 0.245*** | 0.177*** |
|  | (0.005) | (0.005) | (0.006) | (0.012) |
| Innovation | 0.0133*** | 0.0163*** | 0.015*** | 0.019* |
|  | (0.004) | (0.005) | (0.005) | (0.010) |
| R&D share | 0.0021*** | 0.002*** | 0.003*** | 0.003*** |
|  | (0.001) | (0.001) | (0.000) | (0.001) |
| Investment | 0.0125*** | 0.0153*** | 0.024*** | 0.023** |
|  | (0.005) | (0.005) | (0.005) | (0.010) |
| ROA | 0.125 | -0.026 | -0.004 | 0.068 |
|  | (0.195) | (0.045) | (0.011) | (0.090) |
| Vertical int. | 0.009 | 0.0119 | -0.005 | -0.004 |
|  | (0.017) | (0.029) | (0.017) | (0.057) |
| Cost of labor | 0.011 | 0.006 | 0.002 | 0.143 |
|  | (0.029) | (0.046) | (0.004) | (0.094) |
| Sales | -- | -0.007 | -- | 0.003 |
|  | -- | (0.007) | -- | (0.012) |
| Sales growth | -- | -0.005 | -- | -0.004 |
|  | -- | (0.005) | -- | (0.010) |
| Leverage | -- | -0.001 | -- | 0.000 |
|  | -- | (0.0047) | -- | (0.009) |
| Net acc. payable | -- | 0.003 | -- | 0.003 |
|  | -- | (0.022) | -- | (0.041) |
| Bank debt | -- | -0.042* | -- | -0.086* |
|  | -- | (0.024) | -- | (0.050) |
| Constant | -2.93*** | -3.41*** | 0.21*** | 0.14 |
|  | (0.306) | (0.432) | (0.049) | (0.158) |
| Controls | | | | |
| Region | yes | yes | yes | yes |
| Industry | yes | yes | yes | yes |
| Time | yes | yes | yes | yes |
| Region*Time | yes | yes | yes | yes |
| Industry*Time | yes | yes | yes | yes |
| Mundlak | yes | yes | -- | -- |
| Firm FE | -- | -- | yes | yes |
| Observations | 23.932 | 14.318 | 51.688 | 14.318 |
| Pseudo R2 | 0.768 | 0.776 | -- | -- |
| R2 | -- | -- | 0.073 | 0.066 |

*Notes: RE-probit models with Mundlak correction (marginal effects in columns 1-2) and linear probability models with firm and time fixed effects (columns 3-4). The dependent variable is the extensive margin of export (Export). All measures are defined in Appendix. *, **, *** denote, respectively, significance at 10%, 5%, and 1% level. Robust standard errors in parentheses.*

**Table A4. Extensive margins of export by geographical maximum extension**

| Y: | Export(EU) | Export(extraEU) |
| --- | --- | --- |
|  | (1) | (2) |
| Innovation | 0.010*** | 0.021*** |
|  | (0.002) | (0.002) |
| R&D share | 0.002*** | 0.001*** |
|  | (0.000) | (0.000) |
| Investment | 0.007*** | 0.005** |
|  | (0.002) | (0.002) |
| Constant | -3.040*** | -2.068*** |
|  | (0.212) | (0.213) |
| Controls |  |  |
| Region | yes | yes |
| Industry | yes | yes |
| Time | yes | yes |
| Region*Time | yes | yes |
| Industry*Time | yes | yes |
| Mundlak | yes | yes |
| Observations | 14.318 | 14.318 |
| Pseudo R2 | 0.795 | 0.749 |

*Notes: RE-probit models with Mundlak correction (marginal effects in columns 1-2). Untabulated controls follow the specification in Table 1. The dependent variable is the extensive margin of export by maximum geographical extention. Export(EU) is a dummy variable identifying companies exporting (at most) in the EU area, while Export(extraEU) is a dummy variable identifying companies exporting in farer countries. The two dependent variables are defined to be mutually exclusive. All measures are defined in Appendix. *, **, *** denote, respectively, significance at 10%, 5%, and 1% level. Robust standard errors in parentheses.*

**Table A5. Extensive margins of export: heterogeneities by type of innovation**

| Y: | Export | | | |
| --- | --- | --- | --- | --- |
| Sample: | Entire | | Previously non exporting | |
|  | (1) | (2) | (3) | (4) |
| R&D share | 0.002*** | 0.004*** | 0.002*** | 0.003*** |
|  | (0.000) | (0.000) | (0.000) | (0.001) |
| Product inn. | 0.0121*** | 0.020*** | 0.021*** | 0.040*** |
|  | (0.003) | (0.006) | (0.005) | (0.008) |
| Process inn. | 0.007* | 0.014* | 0.007 | 0.005 |
|  | (0.004) | (0.007) | (0.006) | (0.009) |
| Organiz. inn. | 0.006* | 0.013** | 0.009* | 0.007 |
|  | (0.003) | (0.006) | (0.005) | (0.007) |
| Constant | -3.023*** | 0.227*** | -1.623*** | -0.167*** |
|  | (0.192) | (0.049) | (0.270) | (0.053) |
| Controls |  |  |  |  |
| Region | yes | yes | yes | yes |
| Industry | yes | yes | yes | yes |
| Time | yes | yes | yes | yes |
| Region*Time | yes | yes | yes | yes |
| Industry*Time | yes | yes | yes | yes |
| Mundlak | yes | -- | yes | -- |
| Firm FE | -- | yes | -- | yes |
| Observations | 56.955 | 57.375 | 30.301 | 37.37 |
| Pseudo R2 | 0.762 | -- | 0.551 | -- |
| R-squared | -- | 0.033 | -- | 0.144 |

*Notes: RE-probit models with Mundlak correction (marginal effects in columns 1 and 3) and linear probability models with firm and time fixed effects (columns 2 and 4). Untabulated controls follow the specification in Table 1. The dependent variable is the extensive margin of export (Export). The left panel reports estimates performed on the entire sample, while the right panel refers to the subsample of previously-non-exporting companies in t-1. All measures are defined in Appendix. *, **, *** denote, respectively, significance at 10%, 5%, and 1% level. Robust standard errors in parentheses.*

**Table A6: Extensive margins of export: upgrading strategies**

| Y: | Export | | | |
| --- | --- | --- | --- | --- |
| Estimator: | RE-Probit with Mundlak | | Linear probability model | |
|  | (1) | (2) | (3) | (4) |
| Upgrading | 0.046*** | 0.048*** | 0.078*** | 0.075*** |
|  | (0.004) | (0.004) | (0.007) | (0.007) |
| Innovation | 0.0396*** | 0.0441*** | 0.063*** | 0.062*** |
|  | (0.006) | (0.006) | (0.010) | (0.011) |
| R&D share | 0.002*** | 0.002*** | 0.004*** | 0.004*** |
|  | (0.000) | (0.000) | (0.001) | (0.001) |
| Constant | -3.050*** | -3.619*** | 0.061 | 0.115 |
|  | (0.373) | (0.439) | (0.149) | (0.157) |
| Controls | | |  | |
| Region | yes | yes | yes | yes |
| Industry | yes | yes | yes | yes |
| Time | yes | yes | yes | yes |
| Region*Time | no | yes | no | yes |
| Industry*Time | no | yes | no | yes |
| Mundlak | yes | yes | -- | -- |
| Firm FE | -- | -- | yes | yes |
| Observations | 14.318 | 14.318 | 14.318 | 14.318 |
| Pseudo R2 | 0.771 | 0.783 | -- | -- |
| R2 | -- | -- | 0.055 | 0.077 |

*Notes: RE-probit models with Mundlak correction (marginal effects in columns 1 and 2) and linear probability models with firm and time fixed effects (columns 3 and 4). Upgrading is the change in the number of dynamic strategies (Innovation and R&D) between t-2 and t-1, with the support [-2,+2]. Untabulated controls follow the specification in Table 1. The dependent variable is the extensive margin of export (Export). All measures are defined in Appendix. *, **, *** denote, respectively, significance at 10%, 5%, and 1% level. Robust standard errors in parentheses.*

**Table A7: firms’ exit after 2011**

| Y: | Exit | |
| --- | --- | --- |
|  | (1) | (2) |
| Innovation | -- | -0.085*** |
|  | -- | (0.011) |
| R&D share | -- | -0.001 |
|  | -- | (0.001) |
| Productivity | -0.014** | -0.012** |
|  | (0.004) | (0.005) |
| Constant | -1.806*** | -0.197*** |
|  | (0.323) | (0.052) |
| Controls |  |  |
| Region | yes | yes |
| Industry | yes | yes |
| Time | yes | yes |
| Region*Time | no | no |
| Industry*Time | no | no |
| Mundlak | yes | yes |
| Observations | 4.951 | 4.951 |
| Prseudo R2 | 0.540 | 0.541 |

*Notes: RE-probit models with Mundlak correction (marginal effects). The dependent variable is a dummy measure identifying firms exiting the international markets after 2011 (Exit). Additional regressors (untabulated) follow the specification in Table 1. All measures are defined in Appendix. *, **, *** denote, respectively, significance at 10%, 5%, and 1% level. Robust standard errors in parentheses.*

**Table A8: Intensive margins of export: Heckman-type selection model**

|  |  |  | |  |  | |
| --- | --- | --- | --- | --- | --- | --- |
| Y: | Export share | | Export sales growth | | |  |
|  | (1) | (2) | | (3) | (4) | |
| Productivity | 2.323*** | 2.323*** | | 0.123*** | 0.123*** | |
|  | (0.112) | (0.112) | | (0.011) | (0.011) | |
| Size | 3.831*** | 3.859*** | | 0.147*** | 0.147*** | |
|  | (0.089) | '(0.089) | | (0.009) | (0.009) | |
| Age | 0.153 | 0.117 | | 0.009 | 0.009 | |
|  | (0.129) | (0.128) | | (0.008) | (0.008) | |
| R&D share | 0.962*** | 0.875*** | | 0.046*** | 0.048*** | |
|  | (0.031) | (0.032) | | (0.011) | (0.012) | |
| Innovation | 1.936*** | -- | | 0.077*** | -- | |
|  | (0.260) | -- | | (0.015) | -- | |
| Product inn. | -- | 4.954*** | | -- | 0.099*** | |
|  | -- | (0.326) | | -- | (0.016) | |
| Process inn. | -- | 0.668* | | -- | 0.002 | |
|  | -- | (0.363) | | -- | (0.018) | |
| Organiz. inn. | -- | -0.246 | | -- | 0.033* | |
|  | -- | (0.322) | | -- | (0.0171) | |
| Export sales | -- | -- | | -0.152*** | -0.152*** | |
|  | -- | -- | | (0.006) | (0.006) | |
| Constant | -15.94*** | -16.09*** | | 1.572*** | 1.588*** | |
|  | (1.244) | (1.239) | | (0.111) | (0.111) | |
| Controls |  |  | |  |  | |
| Region | yes | yes | | yes | yes | |
| Industry | yes | yes | | yes | yes | |
| Time | yes | yes | | yes | yes | |
| Heckman correction | yes | yes | | yes | yes | |
| Observations | 67.108 | 67.108 | | 16.954 | 16.954 | |
| Rho (p-value) | 0.000 | 0.000 | | 0.000 | 0.000 | |
| Sigma (p-value) | 0.000 | 0.000 | | 0.000 | 0.000 | |
| Loglikelihood | -201409 | -201295 | | -28788 | -28785 | |

*Notes: Maximum-Likelihood Heckman-type selection model. Untabulated controls follow the specification in Table 1. The dependent variable is the extensive margin of export defined as the share of exported sales (columns 1 and 2) and growth of exported sales (columns 3 and 4). Estimation is performed using a-la Heckman model with a maximum likelihood method. Excluded variables used in the selection equation are the average export propensity of firms within the same stratum of the company, identified at the 2-digit sector and provincial levels. Both variables capture export spillovers from the surrounding environment and are computed excluding the international status of the company considered (i.e., for company i we take the average export propensity for the* $j\neq i$ *companies in the same sector and province). This approach is in the spirit of Kneller and Pisu (2007). All measures are defined in Appendix. *, **, *** denote, respectively, significance at 10%, 5%, and 1% level. Robust standard errors in parentheses.*

**Table A9: Marginal vs. Large exporters**

| Y: | Export share | | | | |
| --- | --- | --- | --- | --- | --- |
| Quantile: | q10 | q25 | q50 | q75 | q90 |
|  | (1) | (2) | (3) | (4) | (5) |
| R&D | 0.047*** | 0.077*** | 0.118*** | 0.174** | 0.089 |
|  | (0.009) | (0.021) | (0.035) | (0.070) | (0.084) |
| Innovation | 0.439*** | 0.467*** | 0.838*** | 0.432 | 0.070 |
|  | (0.127) | (0.151) | (0.292) | (0.508) | (0.880) |
| Productivity | 0.414*** | 0.536*** | 1.906*** | 2.502*** | 2.544*** |
|  | (0.059) | (0.116) | (0.214) | (0.425) | (0.378) |
| Size | 0.869*** | 1.215*** | 4.329*** | 4.651*** | 3.423*** |
|  | (0.066) | (0.190) | (0.107) | (0.296) | (0.301) |
| Constant | -3.488*** | 0.210 | -4.836*** | 20.253*** | 54.684*** |
|  | (0.685) | -1477 | -1851 | -5256 | -4880 |
| Controls |  |  |  |  |  |
| Region | yes | yes | yes | yes | yes |
| Industry | yes | yes | yes | yes | yes |
| Time | yes | yes | yes | yes | yes |
| Region*Time | yes | yes | yes | yes | yes |
| Industry*Time | yes | yes | yes | yes | yes |
| Mundlak | yes | yes | yes | yes | yes |
| Observations | 28.016 | 28.016 | 28.016 | 28.016 | 28.016 |

*Notes: quantile regressions with Mundlak correction. The dependent variable is the intensive margin of export defined as the share of exported sales on total turnover (Export share). The estimation is performed on the subsample of exporters only. Additional regressors (untabulated) follow the specification in Table 1. All measures are defined in Appendix. *, **, *** denote, respectively, significance at 10%, 5%, and 1% level. Robust standard errors in parentheses.*

**Table A10: Overall performance**

| Y: | Overall sales growth | |
| --- | --- | --- |
|  | (1) | (2) |
| Productivity | 0.018** | 0.018** |
|  | (0.009) | (0.009) |
| R&D share | 0.002** | 0.002** |
|  | (0.001) | (0.001) |
| Innovation | 0.023* | -- |
|  | (0.012) | -- |
| Product inn. | -- | 0.018* |
|  | -- | (0.009) |
| Process inn. | -- | 0.013 |
|  | -- | (0.022) |
| Organiz. inn. | -- | 0.030* |
|  | -- | (0.015) |
| Constant | 4.222*** | 4.229*** |
|  | (0.344) | (0.346) |
| Controls |  |  |
| Region | yes | yes |
| Industry | yes | yes |
| Time | yes | yes |
| Firm FE | yes | yes |
| Observations | 16.954 | 16.954 |
| R2 | 0.144 | 0.145 |

*Notes: within estimators with firm and time fixed effects. The dependent variable is the growth rate of total sales. Additional regressors (untabulated) follow the specification in Table 1. All measures are defined in Appendix. *, **, *** denote, respectively, significance at 10%, 5%, and 1% level. Robust standard errors in parentheses.*

**Table A11: Cumulative effects of dynamic strategies: innovation with or without R&D**

| Y: | Export | | | | Export sales growth |
| --- | --- | --- | --- | --- | --- |
| Estimator: | RE-Probit with Mundlak | | Linear probability model | | Within estimator |
|  | (1) | (2) | (3) | (4) | (5) |
| Innovation with R&D | 0.036*** | 0.041*** | 0.055*** | 0.055*** | 0.117*** |
|  | (0.004) | (0.007) | (0.009) | (0.013) | (0.027) |
| Innovation without R&D | 0.010*** | 0.017*** | 0.017*** | 0.025*** | 0.066*** |
|  | (0.003) | (0.004) | (0.006) | (0.006) | (0.021) |
| R&D share | 0.002*** | 0.002*** | 0.003*** | 0.003*** | 0.006*** |
|  | (0.000) | (0.000) | (0.000) | (0.001) | (0.002) |
| Productivity | 0.001 | 0.002 | 0.004 | 0.004 | 0.038** |
|  | (0.001) | (0.001) | (0.004) | (0.004) | (0.018) |
| Constant | -3.021*** | -1.657*** | 0.228*** | -0.169*** | 7.214*** |
|  | (0.193) | (0.270) | (0.049) | (0.053) | (0.571) |
| Controls | | |  | |  |
| Region | yes | yes | yes | yes | yes |
| Industry | yes | yes | yes | yes | yes |
| Time | yes | yes | yes | yes | yes |
| Region*Time | no | yes | no | yes | yes |
| Industry*Time | no | yes | no | yes | yes |
| Mundlak | yes | yes | -- | -- | -- |
| Firm FE | -- | -- | yes | yes | yes |
| Observations | 56.955 | 30.301 | 57.375 | 37.37 | 16.954 |
| Pseudo R2 | 0.764 | 0.776 | -- | -- | -- |
| R2 | -- | -- | 0.033 | 0.144 | 0.184 |

*Notes: RE-probit models with Mundlak correction (marginal effects in columns 1 and 2) and within estimator with firm and time fixed effects (estimates and marginal effects in columns 3, 4, and 5). The dependent variable is the extensive margin of export (Export), a dummy variable identifying exporting companies in columns 1 to 4, or the growth rate of exported value (Export sales growth in column 5). Innovation with or without R&D are dummy variables identifying innovative firms performing or not performing R&D activity. Additional regressors (untabulated) follow the specification in Table 1. All measures are defined in Appendix. *, **, *** denote, respectively, significance at 10%, 5%, and 1% level. Robust standard errors in parentheses.*

1. Production services that are sampled are: distributive trades (6), transportation and storage services (7), information and communication services (9), administrative and support service activities (12). The methodological issues associated with measuring exports of business services are addressed inter alia by Eurostat in the context of World Trade in Services Statistics. See:https://ec.europa.eu/eurostat/statistics-explained/index.php?title=World_trade_in_services&oldid=452146#:~:text=The%20EU%2D28%20is%20the,20.2%20%25)%20of%20global%20imports. [↑](#footnote-ref-1)
2. Notice that the variable Human capital is largely insignificant, possibly because R&D measures are capturing most of its effect. [↑](#footnote-ref-2)
3. The first principal component accounts for 65% of the total variance and loads on all the coefficients unambiguously indicating increases in creditworthiness. [↑](#footnote-ref-3)
